# Neurobehavioral Dimensions of Prader Willi Syndrome: Relationships Between Sleep and Psychosis-Risk Symptoms

**DOI:** 10.3389/fpsyt.2022.868536

**Published:** 2022-04-13

**Authors:** Kathleen P. O'Hora, Zizhao Zhang, Ariana Vajdi, Leila Kushan-Wells, Zhengyi Sissi Huang, Laura Pacheco-Hansen, Elizabeth Roof, Anthony Holland, Ruben C. Gur, Carrie E. Bearden

**Affiliations:** ^1^Department of Psychiatry and Biobehavioral Sciences, Semel Institute for Neuroscience and Human Behavior, University of California, Los Angeles, Los Angeles, CA, United States; ^2^Neuroscience Interdepartmental Program, University of California, Los Angeles, Los Angeles, CA, United States; ^3^Department of Biostatistics, University of California, Los Angeles, Los Angeles, CA, United States; ^4^Department of Psychology and Human Development, Vanderbilt University, Nashville, TN, United States; ^5^Department of Psychiatry, University of Cambridge, Cambridge, United Kingdom; ^6^Department of Psychiatry, University of Pennsylvania and the Penn-Children's Hospital of Philadelphia (CHOP) Lifespan and Brain Institute, Philadelphia, PA, United States; ^7^Department of Psychology, University of California, Los Angeles, Los Angeles, CA, United States

**Keywords:** Prader Willi Syndrome, psychosis, sleep, genetic subtype, cognition, remote assessment, neurogenetic disorders

## Abstract

**Background:**

Prader Willi Syndrome (PWS) is a genetic disorder caused by the absence of expression of the paternal copies of maternally imprinted gene(s) located at 15q11–q13. While the physical and medical characteristics of PWS, including short stature, hyperphagia and endocrine dysfunction are well-characterized, systematic investigation of the long-recognized psychiatric manifestations has been recent.

**Methods:**

Here, we report on the first remote (web-based) assessment of neurobehavioral traits, including psychosis-risk symptoms (Prodromal Questionnaire-Brief Version; PQ-B) and sleep behaviors (Pittsburgh Sleep Quality Index), in a cohort of 128 participants with PWS, of whom 48% had a paternal deletion, 36% uniparental disomy, 2.4% an imprinting mutation and 13% unknown mutation (mean age 19.3 years ± 8.4; 53.9% female). We aimed to identify the most informative variables that contribute to psychosis-risk symptoms. Multiple domains of cognition (accuracy and speed) were also assessed in a subset of PWS participants (*n* = 39) using the Penn Computerized Neurocognitive Battery (Penn-CNB).

**Results:**

Individuals with PWS reported a range of psychosis-risk symptoms, with over half reporting cognitive disorganization (63.1%) and about one third reporting unusual beliefs (38.6%) and/or suspiciousness (33.3%). Subjectively-reported sleep quality, nap frequency, sleep duration, sleep disturbance, and daytime dysfunction were significant predictors of psychosis-risk symptom frequency and severity (all *p's* < 0.029). Sleep disturbance ratings were the strongest predictors of psychosis-risk symptoms. Regarding cognition, individuals with PWS showed the most prominent deficits in accuracy on measures of social cognition involving faces, namely Face Memory, Age Differentiation and Emotion Recognition, and greatest slowing on measures of Attention and Emotion Recognition. However, there were no significant differences in psychosis-risk symptoms or cognitive performance as a function of PWS genetic subtype.

**Conclusions:**

PWS is associated with a high prevalence of distressing psychosis-risk symptoms, which are associated with sleep disturbance. Findings indicate that self/parent-reported neurobehavioral symptoms and cognition can be assessed remotely in individuals with PWS, which has implications for future large-scale investigations of rare neurogenetic disorders.

## Introduction

Prader Willi Syndrome (PWS) is a genetic disorder caused by the absence of expression of the paternal copies of maternally imprinted gene(s) genes located at 15q11–q13 (1). About 70% of people with PWS have a deletion, 25% are caused by maternal uniparental disomy (mUPD) and a minority are due to imprinting defects or translocation in the PWS region. PWS is associated with a number of physical and medical conditions including hypotonia, short stature, endocrine dysfunction and childhood-onset hyperphagia (2). While these aspects of the disorder are now well-characterized, the psychiatric manifestations, which were first reported in the literature not long after the syndrome was first described, have over the last 20 years, increasingly been the focus of systematic investigation. The neurobehavioral phenotype includes intellectual disability, behavioral problems, inattention, affective disturbance and elevated rates of psychosis, the latter being first reported as more common in those with the UPD subtype of PWS compared to the deletion by Boer et al. (3–5).

Large-scale genetic association studies have identified novel schizophrenia-associated recurrent risk loci within the 15q region (6–8), suggesting that genes within the PWS critical region may play a broader role in psychosis susceptibility in the typically developing population. Notably, a recent meta-analysis of data from five studies concluded that those with mUPD are at particularly high risk for psychosis (5). While there was no over-representation of depressive psychosis in those with mUPD, individuals with mUPD also had higher rates of bipolar illness than those with the paternal deletion. In contrast, people with PWS with paternal deletion had lower Verbal IQ scores relative to mUPD cases, suggesting that the discrepancy is not one of general syndrome severity. These findings suggest mUPD as one potential background risk factor indicating enhanced psychosis risk. However, little is known about dimensional phenotypes (i.e., psychotic-like symptoms) that may predict the emergence of psychosis in PWS. Nor are the behavioral correlates of such symptoms well-understood.

There is converging evidence of wide-ranging sleep disturbances in PWS from both human and animal studies. Polysomnographic (PSG) studies of sleep in humans report increased instances of sleep disordered breathing (SDB) and narcoleptic-like traits, including daytime sleepiness and REM abnormalities in children, adolescents, and adults with PWS (9–13). Translational studies indicate genetic mutations in the PWS region in a mouse model lead to the same REM alterations observed in humans with PWS (14). Specifically, in the lateral hypothalamus (LH) of these mice, there are disruptions of orexin neurons, which promote wakefulness, and neurons that release melanin-concentrating hormone (MCH), which promote sleep (15). However, research on the relationship of sleep disturbance to psychiatric manifestations in PWS is scarce, despite the fact that sleep is a phenotype of interest in relationship to psychosis risk. Youth at clinical high risk (CHR) for psychosis have consistently shown elevated rates of a variety of disturbances in sleep quality, continuity, and architecture (16–19). These sleep disturbances are a risk factor for overt psychosis onset, and have been associated with more severe psychosis-risk symptoms, altered brain morphometry, and decreased daytime function in populations at clinical or genetic high-risk of developing psychosis (16, 18, 20–22). In individuals with overt psychosis, sleep disturbance is associated with worse cognitive deficits, quality of life, and more severe positive and negative symptoms (23–25). Interestingly, recent studies reported that Cognitive Behavioral Therapy for Insomnia (CBT-I) not only improved sleep in individuals with psychosis, but also decreased psychosis symptoms, suggesting a bidirectional relationship between sleep and psychosis symptoms (26, 27). For an in-depth summary of the literature on sleep and psychosis, please see Davies et al. (28). While causal mechanisms are still unclear, these findings collectively highlight sleep disruption as not only a prominent psychiatric illness phenotype, but as a potentially important biomarker and treatment target of psychosis risk.

With regard to neurocognition, global cognitive function is typically in the impaired range [average Full Scale IQ of ~60, but highly variable, ranging from 40 to 100 across studies (29, 30)]. Deficits in emotion recognition and social perception (i.e., accurately judging the intentions of others) are particularly prominent in individuals with PWS (31, 32). There is also some evidence that cognition may differ as a function of genetic subtype: Torrado et al. found that <10% of subjects with paternal deletion had Full-Scale IQ (FSIQ) 70 or greater, whereas over 60% of subjects without the deletion subtype had FSIQ of 70 or higher (33).

In the present study, we sought to remotely evaluate neurobehavioral traits potentially associated with psychosis risk, in order to allow for large-scale data collection; determining the feasibility of such assessments is particularly important for rare disorders, where travel to a research site may not be feasible. Specifically, we assessed dimensionally measured psychosis-risk symptoms, subjective sleep disturbance, and multiple domains of cognition in individuals with PWS. We first characterized psychosis-risk symptoms and sleep disturbance in the whole sample and determined which sleep and demographic variables were the most predictive of overall psychosis-risk symptom frequency and severity. Next, we assessed differences in psychosis-risk symptoms as a function of mutation type. Lastly, in a subset of the sample, we assessed neurocognitive predictors of psychosis-risk symptom frequency and severity.

## Materials and Methods

### Study Procedures and Participants

Potential study participants were notified of the online study via the Foundation for Prader-Willi Research (FPWR), and through Facebook pages and other social media. In order to maximize data collection and study participation, our inclusion criteria were broad: (1) Genetic diagnosis of Prader-Willi Syndrome; (2) ages 12–50; (3) able to provide informed consent or assent.

Study procedures were described in an online consent form; parents or legal guardians signed consent, and a simplified assent form was provided for participants with PWS. After consent and assent forms were completed, a link was provided to the online clinical and neurocognitive assessment forms described below. Study participants and parents each received a $20 gift card for participation. All study procedures were approved by the UCLA Institutional Review Board. All research was performed in accordance with relevant guidelines and regulations, in accordance with the ethical principles for medical research in the Declaration of Helsinki.

### Assessments

Online clinical assessments were completed either by self-report or parent-report, depending on the age and abilities of the participant.

#### Demographic Information

Recorded information included age, sex, race/ethnicity, personal education level, parental education level, and type of PWS mutation.

#### Prodromal Questionnaire-Brief Version

A modified parent-report version of the Prodromal Questionnaire [PQ-B (34)], a screening measure for psychosis-risk symptoms, was administered. This measure includes 21 true/false questions about unusual thoughts and experiences, and associated distress regarding these experiences (“When this happens, he/she feels frightened, concerned or it causes problems”, with responses ranging from “strongly disagree” to “strongly agree”). These items are summarized in Table 1. Responses to the true/false questions are summed to create a frequency score, while distress ratings are summed to create a distress score. It has been shown to have good concurrent validity with an interview-based diagnosis of a psychosis risk syndrome (34). Importantly, this measure is designed to assess subtle symptoms of psychosis risk that may present many years before onset of overt illness; It has shown adequate internal reliability, construct validity, and measurement invariance across race/ethnicity and sex, in 9- to 10-year-old children (35). To our knowledge, the PQ-B has not yet been validated for use as a parent-report measure in the PWS population, therefore we conducted a single-factor Confirmatory Factor Analysis (CFA) to determine validity in this population (see Statistical Analysis).

**Table 1:** PQ-B items.

**Item**	**Name**	**Question**
1	Surroundings	Do familiar surroundings sometimes seem strange, confusing, threatening or unreal to your child?
2	Sounds	Has your child heard unusual sounds like banging, clicking, hissing, clapping or ringing in his/her ears?
3	Different	Does your child sometimes say that things that he/she sees appear different from the way they usually do (brighter or duller, larger or smaller, or changed in some other way)?
4	Experiences	Has your child had experiences with telepathy, psychic forces, or fortune telling?
5	Control	Has your child felt that he/she is not in control of his/her own ideas or thoughts?
6	Talk	Does your child have difficulty getting his/her point across, because they ramble or go off the track a lot when they talk?
7	Feelings	Does your child have strong feelings or beliefs about being unusually gifted or talented in some way?
8	Watching	Does your child feel that other people are watching him/her or talking about him/her?
9	Skin	Does your child complain that he/she sometimes get strange feelings on or just beneath his/her skin, like bugs crawling?
10	Distracted	Does your child sometimes feel suddenly distracted by distant sounds that he/she is not normally aware of?
11	Force	Has your child told you that he/she had the sense that some person or force is around him/her, although he/she couldn't see anyone?
12	Worry	Does your child worry at times that something may be wrong with his/her mind?
13	Exist	Has your child ever said that he/she feels that he/she does not exist, the world does not exist, or that he/she is dead?
14	Confused	Has your child been confused at times about whether something he/she experienced was real or imaginary?
15	Beliefs	Does your child hold beliefs that other people would find unusual or bizarre?
16	Body	Does your child feel that parts of his/her body have changed in some way, or that parts of his/her body are working differently?
17	Thoughts	Does your child ever say that his/her thoughts are sometimes so strong that he/she can almost hear them?
18	Suspicious	Does your child find himself/herself feeling mistrustful or suspicious of other people?
19	Unusual	Has your child seen unusual things like flashes, flames, blinding light or geometric figures?
20	See	Has your child seen things that other people can't see or don't seem to see?
21	Understand	Do people sometimes find it hard to understand what your child is saying?

#### Sleep

We measured subjective sleep quality, sleep duration, sleep latency (i.e., the amount of time it takes to fall asleep), subjective sleep disturbance, daytime dysfunction due to sleepiness, nap frequency, nap duration, sleep consistency, sleep satisfaction, and sleep timing using the Pittsburgh Sleep Quality Index [PSQI (36)], and the RU-SATED, which stands for sleep regularity, satisfaction, timing, duration, efficiency, and alertness during the day (37). Previous studies in children, with and without neurodevelopmental disorders, have used parent-reported sleep questionnaires as a reliable sleep measure, as they have shown to be concordant with objective sleep measures derived from by actigraphy and PSG (38–40).

Higher scores on the PSQI have been associated with increased positive and negative symptom severity in youth at clinical high risk for psychosis (16, 20, 41), suggesting it is a valid measure to address our scientific questions. The PSQI consists of questions requiring subjective ratings from 0 to 3, with higher scores indicating more severe sleep problems, and items requiring time estimates related to sleep onset, sleep duration, and time spent in bed. In the present study, we used the time estimates reported on the PSQI to quantify sleep duration and sleep latency (36), and an additional time estimate of nap duration, which was added by study investigators. Subjective ratings were used to assess sleep quality, sleep disturbance, daytime dysfunction on the PSQI, as well as a nap frequency variable. Subjective ratings on the RU-SATED were used to measure sleep consistency, sleep satisfaction, and sleep timing. See Supplementary Table 1 for a summary of each sleep variable.

#### Penn Computerized Neurocognitive Battery

We employed an online neurocognitive battery that has been extensively validated across a wide age range, which assesses multiple domains of cognition, specifically measures of executive function and attention, verbal and non-verbal memory, verbal and non-verbal reasoning, social cognition (emotion recognition, age differentiation), and processing speed [motor praxis (42, 43)]. The battery has been used in children with intellectual disability (44), captures both accuracy and response time, and employs automated quality assurance and scoring procedures (43).

### Statistical Analysis

To our knowledge, the PQ-B has not yet been validated for use as a parent-report measure in the PWS population. To assess the measure's validity in this population, we first conducted a single-factor Confirmatory Factor Analysis (CFA) on the PQ-B frequency and distress scores. We used the Comparative Fit Index (CFI), Tucker-Lewis index, and root mean-squared error of the approximation (RMSEA) to determine if each model was a good fit, as in Fonseca-Pedrero et al. (45). A CFI and Tucker-Lewis Index value of 0.95 or higher and a RMSEA of 0.08 or less would indicate an acceptable fit for the PQ-B measure to be considered valid (46–48).

Next, we assessed psychosis-risk symptoms, sleep, and neurocognitive abilities in our sample of individuals with PWS. We analyzed the frequency and distribution of all PQ-B symptoms and sleep problems reported. For the neurocognitive domains, accuracy and speed scores on each of the individual neurocognitive measures (Face Memory, Word Memory, Attention Continuous Performance Test, Penn Exclusion Test, Matrix Analysis, Logical Reasoning, Emotion Recognition and Age Differentiation) were transformed to *z*-scores based on the means and standard deviations from a large normative cohort; the Motor Praxis test assessed motor speed with no accuracy score [*n* = 8,739; Philadelphia Neurodevelopmental Cohort (43, 49)].

Next, we sought to determine potential sleep, neurocognitive, genetic, and demographic predictors of psychosis-risk symptoms. Primary predictors of interest included sleep variables reported on the PSQI and RU-SATED (nap frequency, nap duration, sleep quality, sleep duration, sleep latency, sleep disturbance, daytime dysfunction, sleep consistency, sleep satisfaction, and sleep timing) as well as demographic variables (parental education, age, sex). We conducted Spearman correlations between the predictors and the distress score for each individual PQ-B item. If the participant did not endorse the item, they were given a distress score of 0. We then assessed the effect of the predictors of interest on PQ-B frequency and distress scores. If a predictor was found to be significantly correlated with a PQ-B item after False Discovery Rate (FDR) correction (*q* < 0.05), two linear models were conducted to test the effect of the predictor on both PQ-B frequency and distress scores. Age, sex, and reporter (parent vs. self) were added as covariates to these models. The scaled beta coefficients of each predictor were compared to determine the strongest predictors of each outcome (frequency score and distress score). We then conducted a forward stepwise regression in which each predictor was individually added into a model that included the strongest predictor (i.e., the predictor with the largest regression coefficient) and covariates. Models with and without the additional predictor were compared using an ANOVA to determine if addition of the predictor explained additional variance. If addition of the predictor explained significantly more variance (*p* < 0.05), it remained in the model. The stepwise regression procedure was conducted to build models for predicting both PQ-B frequency and distress scores. Since beta coefficients were used to compare prediction ability of each variable, we did not FDR-correct *p*-values of regression models. A secondary, exploratory analysis utilized the same procedure in the subgroup of participants who completed the neurocognitive battery to investigate neurocognitive predictors of psychosis in PWS. However, we assessed correlations between neurocognitive domains and PQ-B frequency and distress score, instead of each individual PQ-B item, due to the small sample size and reduced statistical power for this analysis.

To assess genetic predictors of psychotic experiences, we compared PQ-B frequency and distress scores between patients with and without mUPD. Given the prior literature indicating higher rates of psychotic disorder in PWS patients with mUPD (5), we hypothesized that mUPD patients would endorse increased severity of psychosis-risk symptoms [i.e., attenuated (prodromal) features of conceptual disorganization, perceptual anomalies and unusual thought content]. We conducted multiple linear regression models with PQ-B frequency and distress score as the dependent variable and mutation type as the independent variable. All models controlled for age, sex, and reporter (self vs. parent). Participants with an unknown mutation type were excluded from these analyses.

## Results

Of the 128 respondents that completed demographic information, 100 additionally completed sleep questionnaires and 84 completed the PQ-B (see participant demographics, Table 2). All participants with PQ-B data available also had sleep and demographic measures available for analysis. Supplementary Table 2 summarizes demographic information for this subsample. Of the 84 participants with PQ-B, demographic, and sleep data, 72 (85.7%) of those were parent-reported and 12 (14.3%) were self-reported. A subset of participants (*n* = 40) completed the neurocognitive assessment battery. A summary of their demographic information is presented in Supplementary Table 3. Of the 40 participants who completed neurocognitive assessments, PQ-B data were available for 32 of them. Demographic information of each genetic subtype is presented in Supplementary Table 4.

**Table 2:** Participant demographics.

	**PWS Participants (*n* = 128)**
Age in years (SD)	19.3 (8.4)
Age range in years	10–49
Females, *N* (%)	69 (53.9%)
Ethnicity, *N* (%)	Non-hispanic white = 113 (88.3%)
	Native American = 3 (2.3%)
	African American = 1 (0.8%)
	Asian American = 4 (3.1%)
	Latino/Hispanic = 8 (6.3%)
	Mixed Race/Other = 7 (5.5%)
Highest parental education in years (SD)	15.9 (2.3)
Genetic subtype, *N* (%)	Paternal deletion = 62 (48.4%)
	mUPD = 46 (35.9%)
	Imprinting = 3 (2.2%)
	Unknown = 17 (13.3%)

*mUPD, maternal uniparental disomy*.

### Summary of Psychosis-Risk Symptoms on PQ-B

Individuals with PWS endorsed a range of psychosis-risk symptomatology (PQ-B Frequency Score: Mean = 4.18 ± 4.20; PQ-B Distress Score: Mean = 14.30 ± 17.04). 13.1% (*n* = 11) scored higher than the standardized clinical cut-off score [i.e., >9 points (45)]. The frequency of each item is presented in Table 3. The most frequently endorsed items were those related to cognitive disorganization (Understand: *n* = 53; 63.1%; Talk*: n* = 43; 51.2%), suspiciousness (Watching: *n* = 28, 33.3%; Suspicious, *n* = 19, 22.6%), and unusual beliefs (Control: *n* = 24, 38.6%; Force: *n* = 15, 17.9%; Beliefs, *n* = 23, 27.4%). Existential fears (Exist) and visual perceptual anomalies (Unusual) were relatively uncommon, endorsed by <5% of the sample (*n* = 2 and 4, respectively).

**Table 3:** Frequency of PQ-B items endorsed in all PWS subjects and subjects with and without a mUPD mutation.

**PQ-B item**	**PWS overall** **(*n* = 84)**	**Non-mUPD^+^** **(*n* = 39)**	**mUPD^+^** **(*n* = 32)**
Surroundings *n*, (%)	10 (11.9)	6 (15.4)	2 (6.2)
Sounds *n*, (%)	14 (16.8)	8 (20.5)	4 (12.5)
Different *n*, (%)	12 (14.3)	8 (20.5)	2 (6.2)
Experiences *n*, (%)	9 (10.7)	6 (15.4)	2 (6.2)
Control *n*, (%)	24 (28.6)	13 (33.3)	9 (28.1)
Talk *n*, (%)	43 (51.2)	19 (48.7)	18 (56.2)
Feelings *n*, (%)	16 (19.0)	6 (15.4)	8 (25.0)
Watching *n*, (%)	28 (33.3)	14 (35.9)	9 (28.1)
Skin *n*, (%)	6 (7.1)	4 (10.3)	1 (3.1)
Distracted *n*, (%)	16 (19.0)	10 (25.6)	4 (12.5)
Force *n*, (%)	15 (17.9)	9 (23.1)	4 (12.5)
Worry *n*, (%)	12 (14.3)	6 (15.4)	5 (15.6)
Exist *n*, (%)	2 (2.4)	1 (2.6)	1 (3.1)
Confused *n*, (%)	18 (21.4)	9 (23.1)	8 (21.9)
Beliefs *n*, (%)	23 (27.4)	13 (33.3)	98 (25.0)
Body *n*, (%)	7 (8.3)	3 (7.7)	2 (6.2)
Thoughts *n*, (%)	9 (10.7)	5 (12.8)	3 (9.4)
Suspicious *n*, (%)	19 (22.6)	12 (30.8)	4 (12.5)
Unusual *n*, (%)	4 (4.8)	3 (7.7)	0 (0.0)
See *n*, (%)	11 (13.1)	9 (23.1)	0 (0.0)
Understand *n*, (%)	53 (63.1)	24 (61.5)	21 (65.6)

*mUPD, maternal uniparental disomy*.

*^**+**^13 participants with an unknown mutation type were excluded*.

### Validity of PQ-B in PWS

We performed a single-factor CFA on PQ-B frequency and distress items, as in Fonseca-Pedrero et al. (45). The model fit for frequency items had a Tucker-Lewis Index of 0.978, a CFI of 0.980 and a RMSEA of 0.063. The model fit for distress items had a Tucker-Lewis Index of 0.980, a CFI of 0.982 and a RMSEA of 0.073. These results are summarized in Table 4. The model fit for both the frequency and distress items met standards for a good fit, suggesting that the use of the PQ-B as either a parent or self-report questionnaire in the PWS population is valid.

**Table 4:** Model fit parameters for CFA performed on PQ-B frequency and distress scores.

	**Tucker-lewis index**	**CFI**	**RMSEA**
PQ-B frequency	0.978	0.980	0.063
PQ-B distress	0.980	0.982	0.073

*CFI, comparative fit index; RMSEA, root mean squared error of the approximation*.

### Summary of Subjective Sleep

Of the 100 respondents who completed sleep questionnaires, the data indicated 45.0% of respondents are rarely or only sometimes satisfied with their/their child's sleep, 57.0% of participants take a nap at least once a week, 11.0% of participant's sleep quality is described as bad or very bad, and 14.0% of participants had trouble sleeping in the past month because they cannot breathe comfortably. 17.0% of the sample took either over-the-counter or prescription sleep aids, with 13% taking sleep aids at least once a week, and 10% more than twice per week. Supplementary Tables 2, 3 include a break-down of the frequency of sleep aid use. The mean sleep onset latency was 22.07 ± 70.01 min and mean daytime dysfunction score was 2.45 ± 2.04, indicating mild daytime dysfunction, on average.

### Demographic and Sleep Predictors of Psychosis-Risk Symptoms

**Figure 1:**
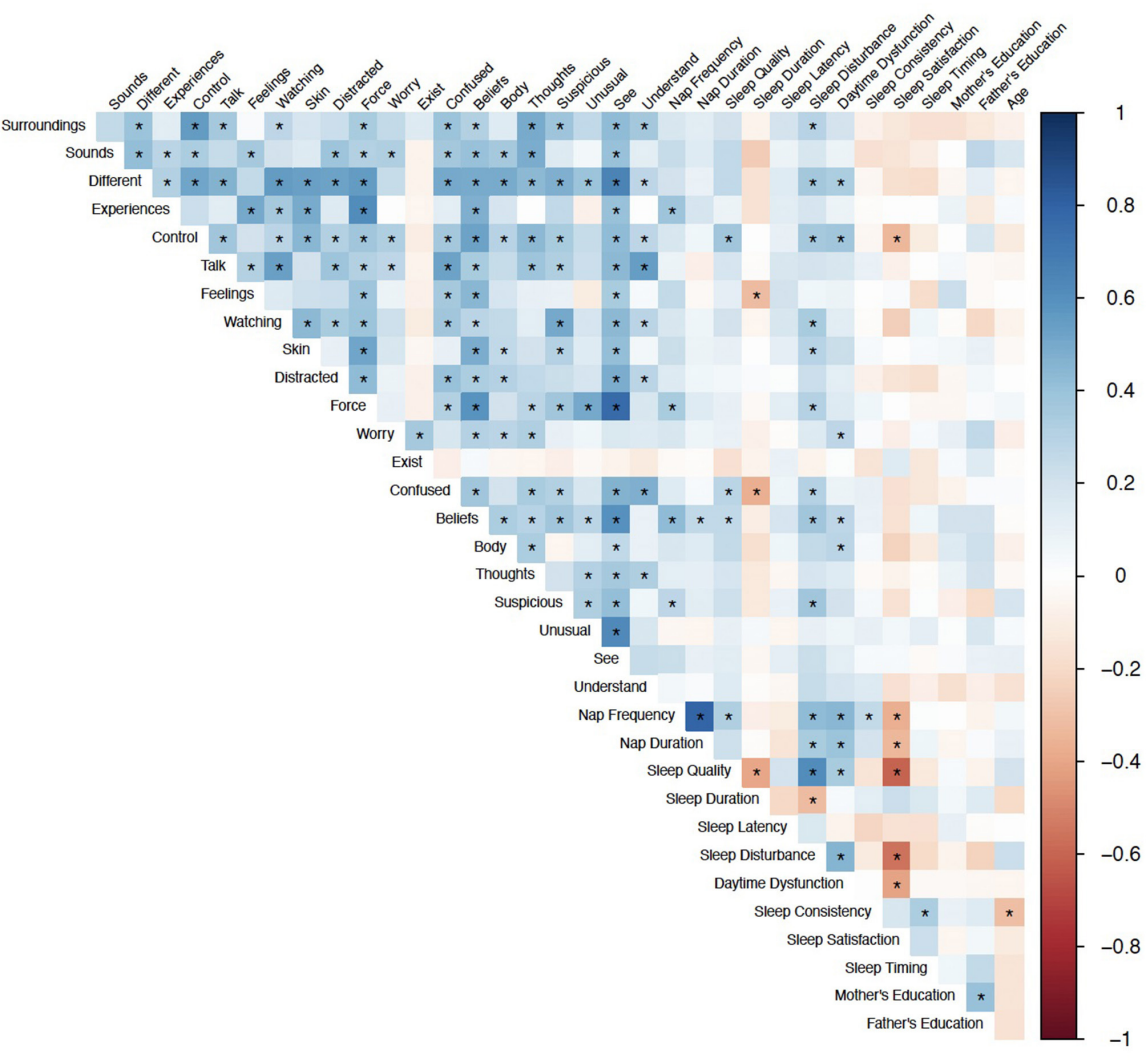
Spearman Correlations between sleep and demographic predictors and PQ-B items. Color indicates correlation coefficient, *Indicates *q* < 0.05.

**Figure 2:**
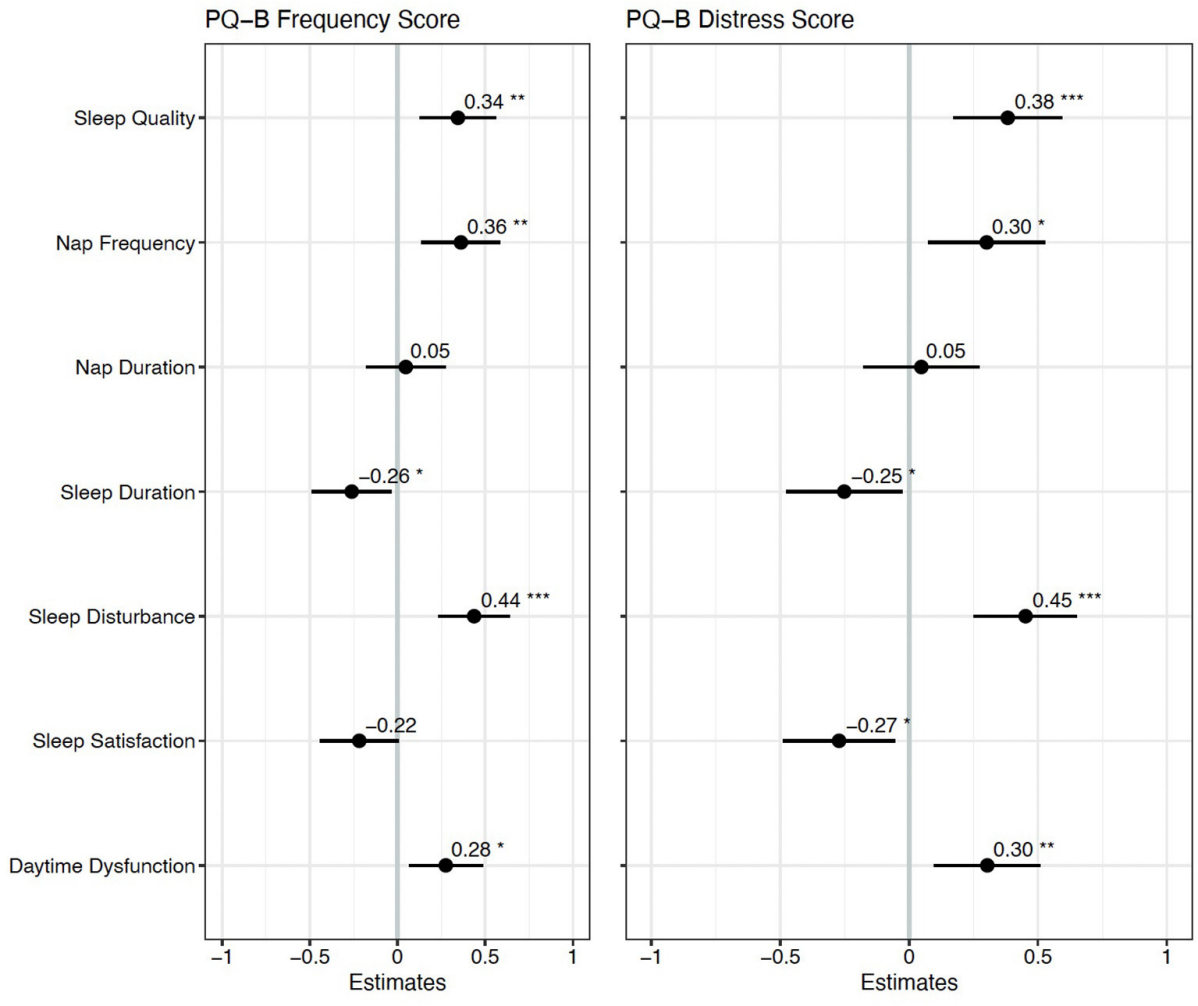
Standardized regression coefficients for each sleep predictor of PQ-B frequency and distress scores. *Indicates *p* < 0.05, **Indicates *p* < 0.01 and ***Indicates *p* < 0.001. Higher frequency and distress scores on the PQ-B were associated with worse sleep quality, more frequent naps, shorter sleep duration, and greater sleep disturbance and daytime dysfunction.

Spearman correlations revealed 24 statistically significant associations between PQ-B items and sleep measures that survived FDR correction. The results are presented in Figure 1. There were no significant correlations between demographic variables (age, mother's education, and father's education) and PQ-B items. Sleep disturbance was positively associated with nine items on the PQ-B (surroundings, different, control, watching, skin, force, confused, beliefs and suspicious; see Table 5), which was the most out of all sleep predictors. Sleep quality, nap frequency, sleep duration, sleep disturbance, and daytime dysfunction were all significant predictors of PQ-B frequency and distress scores in separate regression models (all *p*'s < 0.029; Figure 2). Sleep satisfaction was a significant predictor of distress scores (*p* < 0.016 Figure 2), but only a marginally significant predictor of PQ-B frequency scores (*p* > 0.056). For both frequency and distress scores, sleep disturbance was the strongest predictor (*p*'s < 0.001; Figure 2). There were no significant effects of age or sex on PQ-B frequency or distress scores in these models (all *p*'s > 0.067).

**Table 5:** Significant correlations (*q* < 0.05) between sleep variables and psychosis-risk symptoms measured on the PQ-B.

**Sleep item**	**PQ-B item**	**Correlation coefficient**	***q*-value^**+**^**
Sleep disturbance	Surroundings	0.296	0.025
	Different	0.365	0.004
	Control	0.358	0.004
	Watching	0.341	0.008
	Skin	0.299	0.023
	Force	0.307	0.019
	Confused	0.303	0.021
	Beliefs	0.380	0.003
	Suspicious	0.372	0.003
Sleep duration	Feelings	−0.318	0.014
	Confused	−0.368	0.004
Daytime dysfunction	Difference	0.339	0.008
	Control	0.375	0.003
	Worry	0.274	0.042
	Body	0.290	0.030
Sleep quality	Control	0.376	0.003
	Confused	0.284	0.034
	Beliefs	0.266	0.049
Sleep satisfaction	Control	−0.339	0.008
Nap frequency	Experiences	0.387	0.002
	Force	0.349	0.006
	Beliefs	0.423	< 0.001
	Suspicious	0.279	0.038
Nap duration	Beliefs	0.270	0.046

*PQ-B, prodromal questionnaire-brief version*.

*^+^FDR-corrected p-value*.

Two forward step-wise regression analyses were performed with sleep disturbance, sleep duration, daytime dysfunction, sleep quality, sleep satisfaction, nap frequency, and nap duration as predictors of PQ-B frequency and distress scores. For both PQ-B frequency and distress scores, we began with a model including sleep disturbance as a predictor and covariates of age, sex, and reporter (parent vs. self). No variable explained significantly more variance in PQ-B frequency or distress scores than sleep disturbance did (all *p*'s > 0.122). The final model for PQ-B frequency score, which only included sleep disturbance, had an adjusted *R*^2^ = 0.171 and mean squared error (MSE) = 0.790. The final model for PQ-B distress score, which similarly only included sleep disturbance as a predictor, had an adjusted *R*^2^ = 0.205 and an MSE = 0.757. In the final models, there was a trend toward a significant effect of age on PQ-B frequency score (*b* = −0.211, *p* = 0.067) and distress scores (*b* = −0.188, *p* = 0.094), but no statistically significant effect of sex (all *p*'s > 0.121).

### Relationship Between PWS Genetic Subtype and Reported Psychosis-Risk Symptoms

There were no significant differences in PQ-B frequency (*b* = −0.228, *p* = 0.381) or distress (*b* = −0.319, *p* = 0.221) scores as a function of PWS genetic subtype (mUPD vs. other genetic subtype). These results remained similar when excluding subjects with an imprinting mutation (see Supplementary Material). The most frequently items endorsed on the PQ-B for participants with an mUPD mutation were cognitive symptoms (Understand: *n* = 21, 65.6%; Talk: *n* = 18, 56.2%), as well as unusual thought content (Control: *n* = 9, 28.1%; Beliefs: *n* = 8, 25.0%), and suspiciousness (Watching: *n* = 9, 28.1%). These results are summarized in Table 3. We ran the previously identified “best fit” models for PQ-B frequency and distress scores on only participants with an mUPD mutation and compared the model fit with that of the whole sample. The model performed slightly better in predicting PQ-B frequency and distress scores in the mUPD group as compared to the whole sample. Comparison of the model fits are summarized in Table 6.

**Table 6:** Comparison of model fit between whole sample and mUPD subjects only.

	**Whole sample (*****n*** **=** **84)**		**mUPD subjects only (*****n*** **=** **32)**	
	**MSE**	***Adjusted R*** **^2^**	**MSE**	***Adjusted R*** **^2^**
PQ-B frequency score	0.790	0.171	0.737	0.127
PQ-B distress score	0.757	0.205	0.693	0.155

*MSE, mean squared error; mUPD, maternal uniparental disomy*.

### Neurocognitive Profile

**Figure 3:**
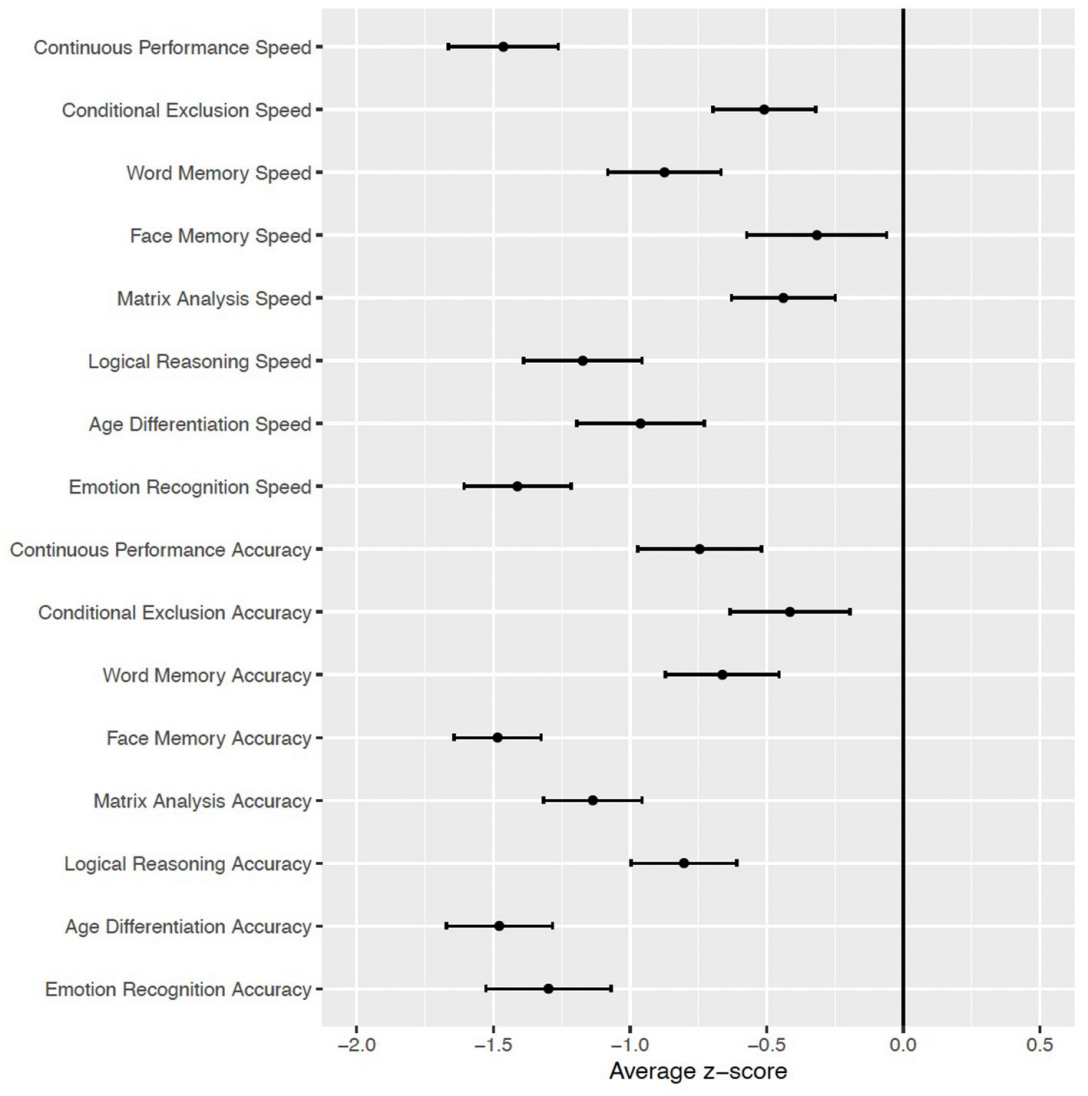
Mean and standard error of neurocognitive domains in subjects with PWS (*n* = 40), relative to typically developing youth cohort [Philadelphia Neurodevelopmental Cohort (49)].

Compared to the cohort of typically developing individuals (49), participants with PWS showed generalized deficits in accuracy, with effect sizes ranging from moderate to large (>1 standard deviation below normative values). The deficits in accuracy were especially pronounced for tasks involving faces: namely, Face Memory and measures of social cognition involving faces (Age Differentiation and Emotion Recognition; Figure 3). With regard to speed of performance, those with PWS performed more slowly than typically developing youth, with the greatest deficits in Attention (Continuous Performance; *z* = −1.5 ± 0.20) and Emotion Recognition (*z* = −1.4 ± 0.21; see Figure 3). In secondary analyses investigating cognitive deficits as a function of mutation type (mUPD *n* = 14; Paternal deletion *n* = 20; Imprinting mutation, *n* = 1; Unknown, *n* = 5), there were no differences between groups as a function of mutation type that survived multiple comparison correction (all *q*'s >0.238).

### Neurocognitive Predictors of Psychosis-Risk Symptoms

**Figure 4:**
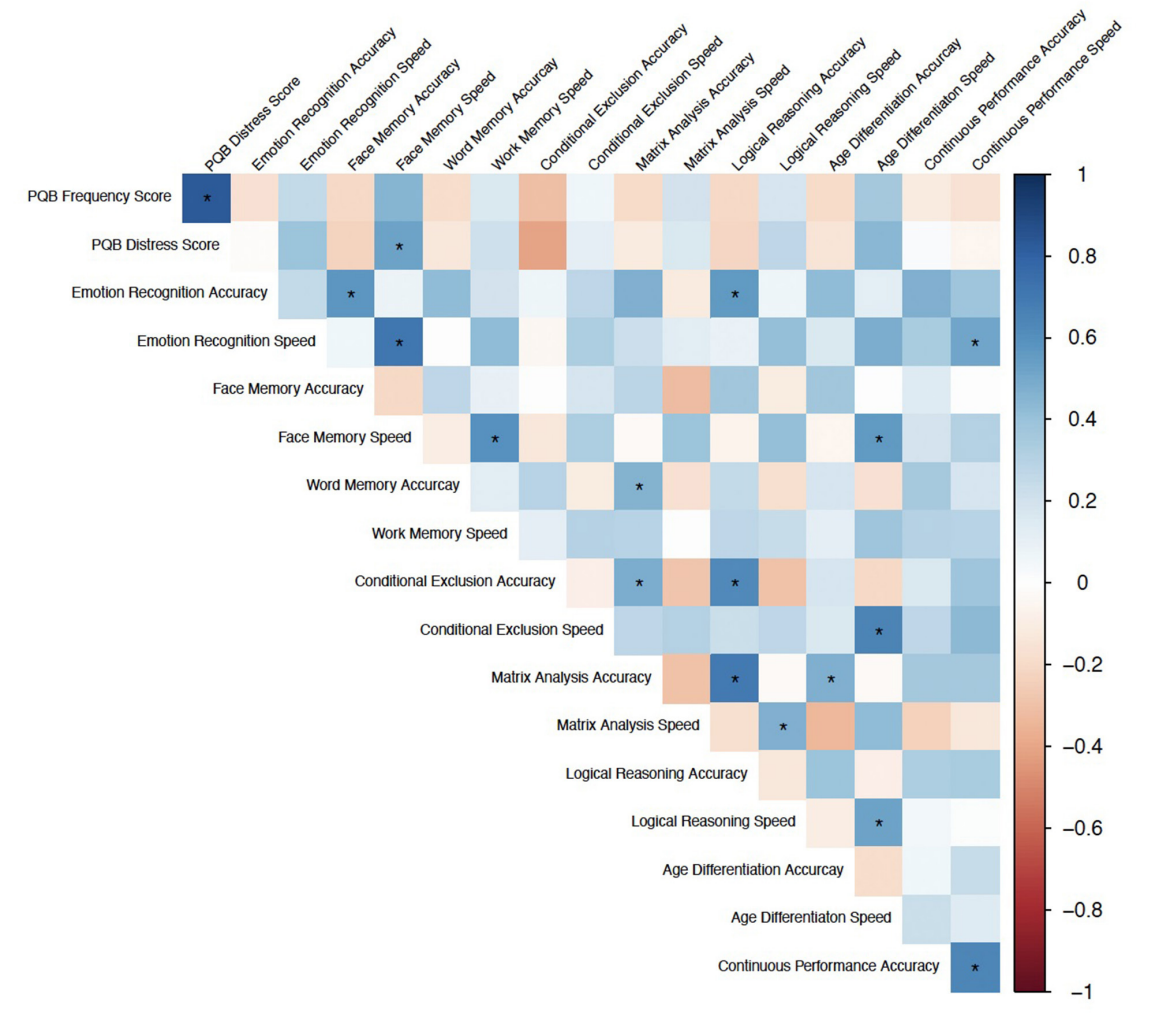
Spearman Correlations between neurocognitive domains (accuracy and speed) and PQ-B frequency and distress scores. Color indicates correlation coefficient, *Indicates *q* < 0.05.

There was a significant correlation between Face Memory Speed, a measure of episodic memory and social cognition, and PQ-B distress score (*r* = 0.529, *q* = 0.021; Figure 4). No other correlations between neurocognitive measures and PQ-B frequency and distress scores survived FDR correction at *q* < 0.05. In regression analyses controlling for age, sex, and reporter, overall, Face Memory speed was a significant predictor of PQ-B frequency and distress scores (*b* = 0.506, *p* = 0.004 and *b* = 0.496, *p* = 0.004, respectively), with increased speed indicating worse symptom scores. Further, the addition of Face Memory Speed into the previously determined “best-fit” model explained significantly more variance in both PQ-B frequency and distress scores (Frequency: F_(26, 1)_ = 14.872, *p* < 0.001; Distress: F_(26, 1)_ = 21.268, *p* < 0.001) in the subset of the sample who completed both the PQ-B and neurocognitive measures (*n* = 32). Therefore, the strongest overall predictors for PQ-B frequency and distress scores in our sample of subjects with PWS were sleep disturbance and Face Memory Speed. The model including Face Memory Speed and sleep disturbance had an adjusted *R*^2^ = 0.400 and MSE = 0.496 for predicting frequency, and adjusted *R*^2^ = 0.501 and MSE = 0.409 for predicting distress score.

## Discussion

To our knowledge, this is the first remote assessment of neurobehavioral traits (psychosis-risk symptoms and sleep) and neurocognition in individuals with PWS. These results are important as they show, in a relatively large sample, a substantial proportion of individuals with PWS experience both distressing psychosis-risk symptoms and disrupted sleep. The most frequently endorsed symptoms in individuals with PWS were related to cognitive disorganization, endorsed in about half the sample, and suspiciousness and unusual beliefs, endorsed in about one-third of the sample. Regarding relationships between sleep disturbance, as measured by the PSQI, and psychosis-risk symptoms, as assessed by the PQ-B, both of the methods we employed revealed that questions on sleep behavior/habits and daytime dysfunction are relatively important for explaining distress related to unusual experiences and other psychosis-risk symptomatology. While excessive daytime sleepiness is a common, well-established phenomenon in individuals with PWS, related to hypothalamic dysfunction (15, 50), our findings also show it is associated with psychosis-risk symptoms. We found that sleep disturbance scores on the PSQI, which includes questions relating to snoring, nighttime awakenings, and nightmares, was the strongest predictor of psychosis-risk symptomatology in individuals with PWS. This could be due to the wide range of sleep phenotypes that have been associated with PWS, including SDB and hypersomnia (11). It is possible that the sleep disturbance measure captures more dimensions of sleep than other measures, such as sleep duration or sleep latency, that could be contributing to psychotic-like symptomatology in individuals with PWS. While sleep research in schizophrenia has focused primarily on thalamocortical dysfunction as a potential mechanism (51–53), it is possible that hypothalamic dysfunction - a hallmark of PWS (15, 54) - could contribute to both the distinct sleep and psychosis phenotype. The hypothalamus is implicated in many of the pathophysiologic processes believed to be relevant to psychosis, including the sleep-wakefulness cycle, hypothalamus-pituitary- gonadal axis dysfunction, and neuroimmune dysfunction (55–58). Additionally, there are similar abnormalities in hypothalamic volume reported in both schizophrenia and affective disorders, which could contribute to the affective, cycloid nature of psychosis in PWS (55, 56). Further research utilizing multi-modal sleep measures is required to determine if certain sleep phenotypes (i.e., excessive daytime sleepiness) are more associated with psychosis-risk symptomatology in PWS than others (i.e., sleep duration). While we cannot determine cause and effect in this cross-sectional study, these findings nevertheless suggest that disrupted sleep may be a precursor to psychotic symptomatology. As a potentially modifiable risk factor, this offers important new information. Other demographic variables, such as age, sex, and parental education level were not as strongly associated with psychosis risk.

This novel web-based approach offers substantial cost savings and efficiency, as no subject travel was required. Further, it allows families in rural locations to participate, who are otherwise unlikely to be able to take part in this research. The development of more scalable methods for research participation is important not only for rare disorders, but for other populations that lack adequate access, such as families with limited financial resources (59).

In help-seeking youth in the general population, the PQ-B shows good concurrent validity with interview-based diagnoses of a psychosis risk syndrome (34). Our CFA analysis suggests that the PQ-B data collected are a reliable and valid reflection of psychosis-risk symptoms for the present analyses. However, certain highly endorsed PQ-B items in individuals with PWS may be related to global cognitive dysfunction, and thus may not have the same prognostic significance. Further, there are likely other factors relevant to the development of overt psychotic illness in PWS that are not represented in the PQ-B. Due to the cross-sectional nature of the current study we cannot determine the relationship between symptom endorsement on the PQ-B and development of overt psychosis. Longitudinal studies are warranted to evaluate the predictive validity of individual PQ-B items in the PWS population.

Contrary to expectations based on prior reports of higher rates of overt psychotic disorder in PWS resulting from mUPD (3, 60), we did not see a difference in psychosis-risk symptoms as a function of PWS genetic subtype. However, there are a number of reasons this may be the case. First, psychosis-risk symptoms are not equivalent to a psychotic disorder, and prior evidence suggests that psychotic symptoms in individuals with PWS have an uncharacteristically acute onset (61, 62). The phenomenology of psychosis in PWS also is distinct from “typical” psychosis presentation, in that there is a strong affective component and a more “cycloid” pattern (4). This notion is also supported by work in animal models of PWS that reported enrichment for variants associated with psychosis-related episodes, but not variants related to schizophrenia, suggesting there may be a genetic liability for psychosis in PWS, that is distinct from schizophrenia (63). As such, future studies should assess the relationship between affective symptoms and psychosis risk in PWS. Modifications to our questionnaire, based on retrospective studies, may be needed to better address the components of psychosis that are distinct from schizophrenia.

Although our cognitive assessment was only completed by a subset of the larger sample, findings revealed differential impairment in social cognitive domains, particularly tasks involving processing of faces, implicating cortico-limbic dysfunction (64). This pattern of findings is highly consistent with other recent work in individuals with PWS, involving in-lab assessment (32, 65). Another recent study offers a translational perspective on psychiatric manifestations of PWS, through the RDoC matrix (50): deficits in social processes are highlighted, with event-related potential (ERP) evidence that people with PWS have altered processing of faces (66). It has been hypothesized that dysfunctional hypothalamic oxytocin-expressing neurons may underlie these deficits in PWS (67). Interestingly, these deficits - Face Memory speed in particular - were strong predictors of psychosis-risk symptoms in our sample. This is consistent with prior literature reporting hippocampally-mediated deficits in facial memory in schizophrenia and other populations at high-risk for psychosis (68–71). However, these analyses only included a small subset of the overall sample; as such, replication in a larger sample is warranted.

### Limitations

Several limitations of the current study should be noted. First, this may not be a representative sample of people with PWS; for example, those who may have been mentally unwell at the time of the study may not have been able to participate. Further, the fact that some participants self-reported while others did not could have created bias in the sample such that those who self-reported were older or higher functioning. We addressed this concern by including reporter as a covariate in the regression analyses. Secondly, the online format of the study limited the amount of detailed prior history we could obtain on study participants. As such, we were only able to obtain cross-sectional information on current (past month) sleep and psychotic-like experiences and we were not able to collect information on whether there was a previous history of a psychotic illness or sleep disorder that had been treated. We also were unable to collect information of growth hormone therapy (GHT) and body mass index (BMI). Existing data suggests a complex relationship between sleep apnea, GHT, and BMI in PWS (72–77) with some studies showing associations and others not. However, since our analyses studied multiple dimensions of sleep, it is unclear if inclusion of these variables would impact our results. Thirdly, we do not have information on the use of psychiatric medications, which -if being used - could suppress symptoms. Fourthly, we had a more limited number of completers for the neurocognitive assessment (relative to questionnaires), which could have led to inadequate statistical power to detect neurocognitive differences between genetic subtypes. Thus, future research in a larger sample is required to validate our findings on neurocognitive predictors of psychosis-risk symptoms and investigate neurocognitive differences between genetic subtypes of PWS. Finally, given the age range of our study participants at the time of assessment, some were below the expected age for onset of overt psychotic illness; however, it has been noted that the age of onset of psychosis in PWS tends to be earlier than in the general population (78). Moreover, the focus of the PQ-B is on psychosis-risk symptoms, not diagnosis of overt psychotic disorder, and the PQ-B has been shown to be a valid self-report measure in children as young as 10 (35).

## Conclusions

PWS is associated with a high prevalence of distressing psychosis-risk symptoms, which were associated with sleep disturbances. Social cognition, particularly tasks involving faces, showed differential impairment. These results support feasibility of remote assessment of individuals with rare neurogenetic syndromes.

## Data Availability Statement

The raw data supporting the conclusions of this article will be made available by the authors, without undue reservation.

## Ethics Statement

The studies involving human participants were reviewed and approved by UCLA Institutional Review Board. Written informed consent to participate in this study was provided by the participants' legal guardian/next of kin.

## Author Contributions

CB designed the study and drafted the manuscript. KO'H and ZZ conducted statistical analyses and drafted methods and results text. KO'H, ZZ, ZH, and AV contributed to data management, quality control, and analyses. LP-H, LK-W, and AV oversaw recruitment and participant assessment. RG designed the neurocognitive battery and provided critical input on interpretation of results. ER and AH provided critical input on the manuscript text and interpretation of results. All authors participated in manuscript revisions and have given final approval of this version for submission.

## Funding

This work was supported by the Foundation for Prader Willi Research (FWPR; CB) and NIMH MH117014 (RG).

## Conflict of Interest

The authors declare that the research was conducted in the absence of any commercial or financial relationships that could be construed as a potential conflict of interest.

## Publisher's Note

All claims expressed in this article are solely those of the authors and do not necessarily represent those of their affiliated organizations, or those of the publisher, the editors and the reviewers. Any product that may be evaluated in this article, or claim that may be made by its manufacturer, is not guaranteed or endorsed by the publisher.
