# Supplementary material for: Neurobehavioral Dimensions of Prader Willi Syndrome: Relationships Between Sleep and Psychosis-Risk Symptoms
Source: Front Psychiatry. 2022 Apr 13;13:868536. doi: 10.3389/fpsyt.2022.868536 (PMC9043455; doi:10.3389/fpsyt.2022.868536)
Supplement: Supplementary file 1 [file Data_Sheet_1.docx]

Supplementary Material

**Supplementary Table 1.** Summary of Sleep Outcomes of interest.

| **Type** | **Questionnaire** | **Measure** | **Scale** | **Items^+^** |
| --- | --- | --- | --- | --- |
| Subjective Rating | PSQI | Sleep Quality | 0=Very good  1=Fairly good  2=Fairly bad  3=Very bad | During the past month, how would you rate your child’s sleep quality overall? |
|  |  | Sleep Disturbance | 0=Not during the past month  1=Less than once a week  2=Once or twice a week  3=Three or more times a week | During the past month, how often has your child had trouble sleeping because he/she wakes up in the middle of the night or early morning? |
|  |  |  |  | During the past month, how often has your child had trouble sleeping because he/she has to get up to use the bathroom? |
|  |  |  |  | During the past month, how often has your child had trouble sleeping because he/she cannot breathe comfortably? |
|  |  |  |  | During the past month, how often has your child had trouble sleeping because he/she cough or snore loudly? |
|  |  |  |  | During the past month, how often has your child had trouble sleeping because he/she feels too cold? |
|  |  |  |  | During the past month, how often has your child had trouble sleeping because he/she had bad dreams? |
|  |  |  |  | During the past month, how often has your child had trouble sleeping because he/she have pain? |
|  |  |  |  | During the past month, how often has your child had trouble sleeping because of other reasons? |
|  |  | Daytime Dysfunction | 0=Not during the past month  1=Less than once a week  2=Once or twice a week  3=Three or more times a week | During the past month, how often has your child had trouble staying awake while driving, eating meals, or engaging in social activity? |
|  |  |  |  | During the past month, how much of a problem has it been for your child to keep up enough enthusiasm to get things done? |
|  | RU-SATED | Sleep Consistency | 0=Rarely/Never  1=Sometimes 2=Usually/Always | Does your child go to bed and get out of bed at about the same time (within one hour) every day? |
|  |  | Sleep Satisfaction | 0=Rarely/Never  1=Sometimes 2=Usually/Always | Are you satisfied with your child's sleep? |
|  |  | Sleep Timing | 0=Rarely/Never  1=Sometimes 2=Usually/Always | Is your child asleep (or trying to sleep) between 2:00 a.m. and 4:00 a.m.? |
|  | N/A^*^ | Nap frequency | 0=Not during the past month 1=Less than once a week  2=Once or twice a week  3=Three or more times a week | During the past month, has your child taken naps during the day? |
| Time Estimate | N/A^*^ | Nap Duration | Minutes | If you checked Once or twice a week or more, how long do he/she typically nap for? |
|  | PSQI | Sleep Duration | Hours | During the past month, how many hours of actual sleep did your child (you) get at night? (This may be different than the number of hours he/she spent in bed) |
|  |  | Sleep Latency | Minutes | During the past month, how long has it usually taken your child to fall asleep each night? |

^+^Wording of items were changed for participants completing questionnaires on their own behalf

^*^Napping variables were created by investigators

**Supplementary Table 2.** Demographics of participants included in analysis of predictors of Psychosis-Risk Symptoms

|  | **PWS Participants (n = 84)** |
| --- | --- |
| Age in years (SD) | 19.1 (8.5) |
| Age Range in years | 10-49 |
| Females, N (% ) | 50 (59.5%) |
| Ethnicity, N (%) | Non-Hispanic White = 71 (84.5%) |
|  | Native American = 2 (2.4%%) |
|  | African American = 1 (1.2%) |
|  | Asian American = 3 (3.6%) |
|  | Latino/Hispanic = 4 (4.8%) |
|  | Mixed Race/Other = 3 (3.6%) |
| Highest Parental Education in years (SD) | 16.0 (2.2) |
| Genetic Subtype, N (%) | Paternal Deletion = 38 (53.5%) |
|  | Maternal Uniparental Disomy (mUPD) = 32 (45.1%) |
|  | Imprinting = 1 (1.4%) |
|  | Unknown = 13 (15.5%) |
| Sleep Medication Use, N (%) | None = 73 (87.0%) |
|  | Less than Once a Week = 3 (3.6%) |
|  | Once or Twice a Week = 2 (2.4%) |
|  | Three or More Times a Week = 6 (7.1%) |

**Supplementary Table 3.** Demographics of participants who completed Neurocognitive Battery

|  | **PWS Participants (n = 40)** |
| --- | --- |
| Age in years (SD) | 21.2 (10.5) |
| Age Range in years | 10-49 |
| Females, N (% ) | 22 (55.0%) |
| Ethnicity, N (%) | Non-Hispanic White = 36 (90.0%) |
|  | Native American = 2 (5.0%%) |
|  | African American = 0 (0.0%) |
|  | Asian American = 1 (2.5%) |
|  | Latino/Hispanic = 1 (2.5%) |
|  | Mixed Race/Other = 0 (0.0%) |
| Highest Parental Education in years (SD) | 16.0 (2.2) |
| Genetic Subtype, N (%) | Paternal Deletion = 20 (57.1%) |
|  | Maternal Uniparental Disomy (mUPD) = 14 (40.0%) |
|  | Imprinting = 1 (2.9%) |
|  | Unknown = 5 (12.5%) |
| Sleep Medication Use^+^, N (%) | None = 33 (84.6%) |
|  | Less than Once a Week = 2 (5.1%)) |
|  | Once or Twice a Week = 2 (5.1%) |
|  | Three or More Times a Week = 2 (5.1%) |

^+^Data unavailable for one subject (n=39)

**Differences in PQ-B scores between genetic subtypes:**

There was no difference in PQ-B frequency (b=-0.247 , p=0.341) or distress scores (b=-0.340 , p=0.193) between subjects with a mUPD mutation versus those with a paternal deletion (excluding those with imprinting mutations from analysis).

**Supplementary Table 4.** Demographics of sample split by genetic subtype

|  | **Paternal Deletion (n=62)** | **mUPD (n=46)** | **Imprinting (n=3)** |
| --- | --- | --- | --- |
| Age in years (SD) | 17.25(6.8) | 18.0 (8.3) | 36.0 (4.0) |
| Age Range in years | 10-44 | 10-49 | 32-40 |
| Females, N (% ) | 27 (43.5%) | 31 (67.4) | 2 (66.7%) |
| Ethnicity, N (%) |  |  |  |
| Non-Hispanic White | 49 (79.0%) | 39 (84.8%) | 3 (100%) |
| Native America | 1 (1.6%) | 1 (2.2%) | 0 (0.0%) |
| African American | 0 (0.0%) | 0 (0.0%) | 0 (0.0%) |
| Asian American | 2 (3.2%) | 1 (2.2%) | 0 (0.0%) |
| Latino/Hispanic | 4 (6.5%) | 4 (8.7%) | 0 (0.0%) |
| Mixed Race/Other | 6 (9.7%) | 1 (2.2%) | 0 (0.0%) |
| Highest Parental Education in years (SD) | 16.2 (2.52) | 16.3 (1.9) | 13.33 (1.15) |
